# Analysis of root morphology in alfalfa (*Medicago sativa L.*) seedlings cultivated with ammonium or nitrate nitrogen using fractal dimension and lacunarity

**DOI:** 10.1186/s12870-025-07902-y

**Published:** 2025-12-22

**Authors:** Daisuke Hirose

**Affiliations:** Faculty of Environment and Horticulture, MinamiKyushu University, Miyakonojo, Miyazaki, 885-0035 Japan

**Keywords:** Alfalfa, Fractal dimension, Lacunarity, Nitrogen fertilizer, Root system

## Abstract

**Background:**

This study aimed to elucidate in greater detail the relationship between the chemical forms of applied nitrogen and root system development in alfalfa (*Medicago sativa* L.) by applying fractal dimension (D) and lacunarity (Lf) as analytical indicators.

**Results:**

Total root length increased exponentially with plant growth under both ammonium nitrogen (NH₄-N) and nitrate nitrogen (NO₃-N) treatments. After 40 days after sowing (40 DAS), the total root length under NH₄-N treatment became significantly greater than that under NO₃-N treatment. A similar tendency was observed for the number of lateral roots, which was also significantly higher under the NH₄-N treatment than under the NO₃-N treatment after 40 DAS. D increased approximately lineary with plant growth under both nitrogen treatments and was higher under the NH₄-N treatment than under the NO₃-N treatment only at 30 and 40 DAS. Lf increased until 20 DAS under both treatments, then decreased as growth continued. A significantly higher Lf was detected under the NH₄-N treatment than under the NO₃-N treatment at 20 DAS only. Both treatments showed an increasing trend in D in the upper root region as growth progressed. In contrast, in the lower region, D decreased until 20 DAS and then began to increase thereafter. Lf in the upper region reached its highest value at 20 DAS for both nitrogen forms, whereas in the lower region it peaked at 30 DAS. Over the entire growth period, no overall correlation was observed between Lf and D, total root length, or lateral root number; however, stage-specific correlations were evident when analyzed by growth phase.

**Conclusions:**

The application of D and Lf made it possible to identify differences in root system development caused by the chemical forms of applied nitrogen that could not be detected through root length or root number alone. The findings further suggest that the effects of distinct nitrogen forms on root development first emerge in the spatial distribution of roots, then in root system complexity, and ultimately in total root length and lateral root number.

## Background

 Ammonium nitrogen (NH_4_-N) and nitrate nitrogen (NO_3_-N) are important nitrogen (N) sources for crop [1]. It has been reported that applying NH₄-N promotes superior growth of alfalfa (*Medicago sativa* L.) compared with NO₃-N [2, 3]. Differences in plant growth arising from the chemical form of applied N have been partly attributed to variation in N uptake [4]. In addition, comparison of root system length and number—the main organs responsible for N absorption—has shown that NH₄-N application increases both total root length and root number relative to NO₃-N application [3]. However, although N uptake under NH₄-N treatment was higher than that under NO₃-N treatment at 25 days after sowing (25 DAS) [4], no significant differences in total root length or root number were detected between the two treatments until 50 DAS [3]. Variation in root morphology occurs due to genetic and environmental factors influencing root development, elongation, and spatial distribution [5]. Therefore, the mismatch between the timing of changes in root development and that of N uptake is thought to be one reason why root length and number alone cannot fully represent detailed patterns of root system development.

On the other hand, it has been shown that fractal dimension (D), a method of fractal analysis, can quantitatively describe the complexity of root systems beyond what is captured by only considering root length and number [6, 7]. D has been reported to increase with root growth [7–11], and to vary depending on plant species and cultivars [8, 11–14]. However, root systems with similar D values can exhibit markedly different visible morphologies [14, 15]. In such cases, lacunarity (Lf), another parameter derived from fractal analysis, has been shown to distinguish between slight morphological differences more effectively [14, 15]. Therefore, using D and Lf may reveal more key details characterizing root system development.

In this study, NH₄-N or NO₃-N was applied to alfalfa seedlings grown hydroponically, and total root length, lateral root number, D, and Lf were measured. By applying D and Lf, this study aims to elucidate finer differences in root system development arising from the chemical forms of applied N that cannot be clearly identified by root length or root number alone.

## Methods

### Plant variety and cultivation method

 Alfalfa plants were grown in a glasshouse at Minamikyushu University, Japan, using the alfalfa variety ‘Tsuyuwakaba’ as the test. Alfalfa seeds were purchased from Snow Brand Seed Co., Ltd (Sapporo, Japan). Seeds were sown in a plastic container (27 × 34 × 10 cm) filled with vermiculite on October 8, 2009, and were supplied only with distilled water until October 15. On that date, germinated seedlings were transplanted into a larger container (54 × 36 × 25 cm) (35 seedlings per container), and hydroponic culture was started. Hydroponic cultivation and management methods followed previous reports [2, 3].

### Set-up of the experimental treatments

Two treatments were prepared: the NO₃-N treatment, where sodium nitrate was the only N source, and the NH₄-N treatment, where ammonium sulfate was used as the N source (Table 1). Two replicate containers (35 plants per container) were used per treatment, with the same N concentration of 20 mg/L. Other nutrient concentrations are shown in Table 1. Each container received 40 L of nutrient solution, renewed every 5 days [2, 3]. The solution pH was adjusted to 6.5 every morning and evening using 1-mol NaOH or HCl.

**Table 1:** Composition of water culture solution excluding nitrogen

Compound	mg/L
KH_2_PO_4_	43.9
K_2_SO_4_	67.0
CaCl_2_.2H_2_O	368.0
MgSO_4_.7H_2_O	102.0
EDTA-Na-Fe	13.1
MnSO_4_.5H_2_O	1.3
ZnSO_4_.7H_2_O	2.2
H_3_BO_3_	2.8

### Timing of the root system collection

Root systems were sampled every 10 days, six times in total, from 10 DAS to 60 DAS. At each sampling time point, six seedlings were randomly selected from each treatment group (three seedlings per replicate container); these parameters were selected based on previous studies [2, 3]. Moreover, this sample size was sufficient to detect differences in the fractal parameters of alfalfa root system. Root systems were immediately placed in FAA solution [formaldehyde: acetic acid: ethanol: distilled water = 1:1:9:9 (v/v)] and stored in a refrigerator at ~ 5 °C for one month before imaging.

### Measurement method of the root parameters

Roots were dyed using 0.5% Coomassie Brilliant Blue solution for 2 h, as described previously [3], and carefully spread on a scanner bed (Canoscan 5400 F, Canon). Samples were arranged within A4 size (210 × 297 mm) to minimize crossing. Digitized images were saved at 300 dpi in 256-level grayscale. Images were analyzed using Scion Image beta 4.02 to determine total root length and number of lateral root branches [3, 16, 17].

Fractal analysis was performed by importing root images into Image J 1.42 and obtaining D and Lf parameters using FracLac 2.5 Release 1 d [18]. In addition, to further clarify the detailed root structure and distribution patterns, the images were divided into upper and lower sections, and D and Lf were measured separately for each section. FracLac was operated using its default settings. In FracLac, Lf is calculated as the variability of pixel density among boxes of different sizes based on the coefficient of variation in pixel distribution. The detailed procedure for calculating Lf and the corresponding formulas are provided in *FRACTAL FOR IMAGEJ: Using FracLac V2.0 for ImageJ* (chrome-extension://efaidnbmnnnibpcajpcglclefindmkaj/https://imagej.net/ij/plugins/fraclac/fraclac-manual.pdf). Data are shown as mean ± S.E. Significance between N treatments at the same DAS was tested using Tukey’s test.

## Results

**Fig. 1:**
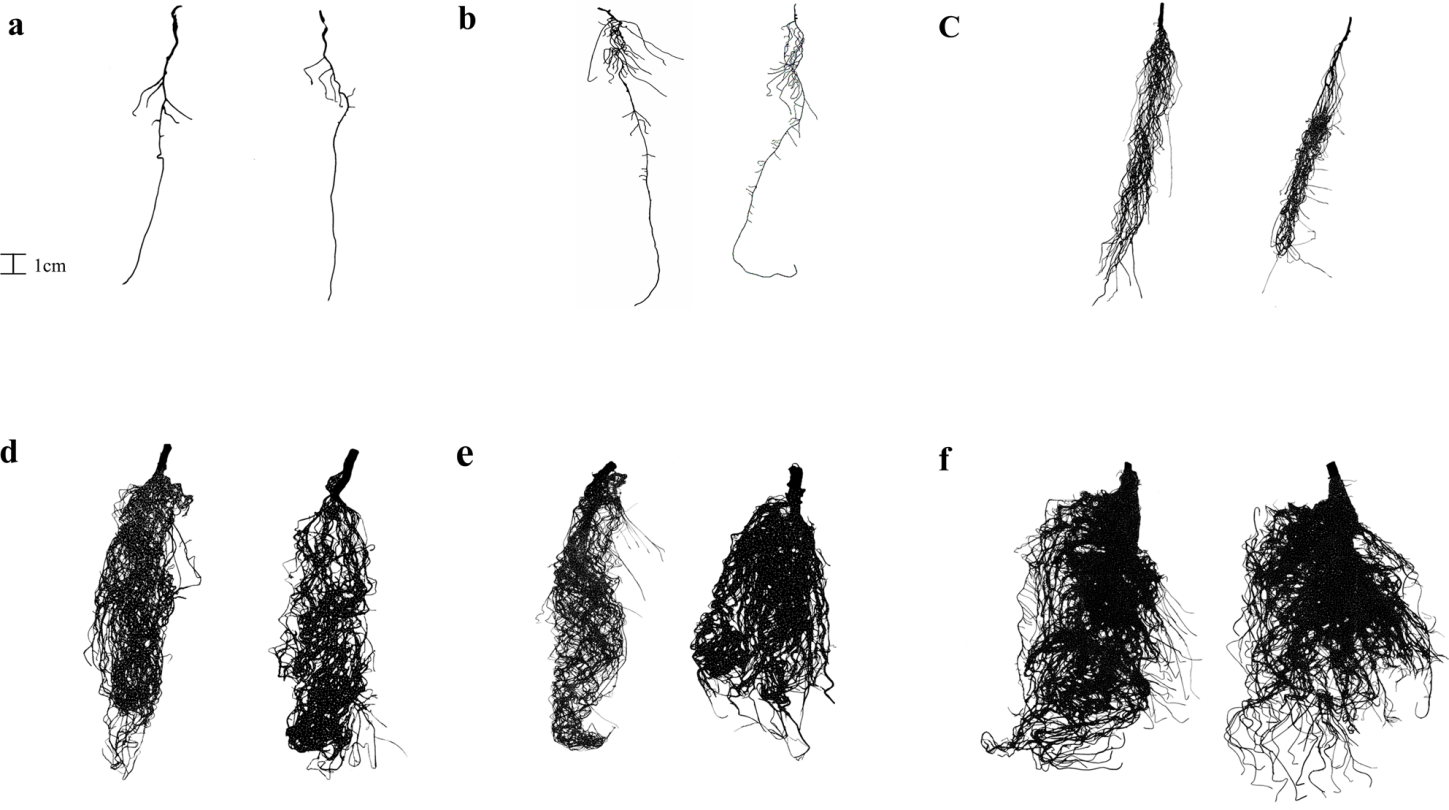
Root images for each treatment. **a**: 10 days after sowing, **b**: 20 days after sowing, **c**: 30 days after sowing, **d**: 40 days after sowing, **e**: 50 days after sowing, **f**: 60 days after sowing. The image on the left at each time point represents the ammonium nitrogen treatment, and the image on the right represents the nitrate nitrogen treatment

Root system images obtained using an image scanner for the NH₄-N and NO₃-N treatments are shown in Fig. 1. In both N treatments, lateral root development was minimal up to 10 DAS. However, from 20 DAS onward, a marked increase in the formation of new lateral roots was observed. Notably, at 50 and 60 DAS, lateral root development had progressed substantially, nearly filling the entire image frame. 

**Fig. 2:**
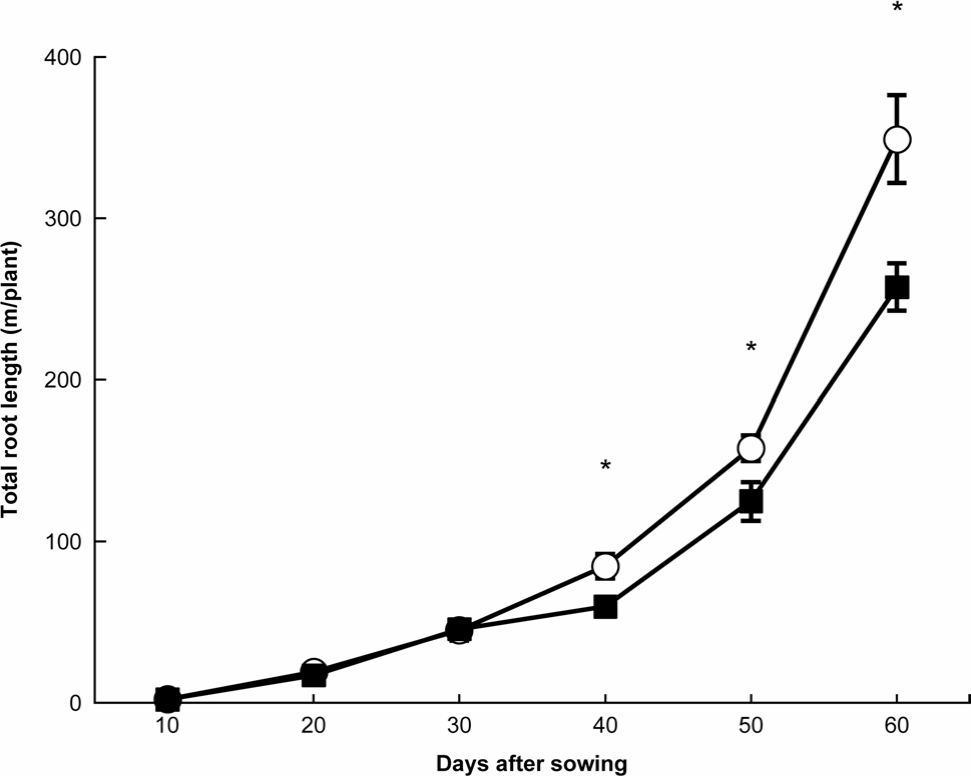
Changes in total root length under each treatment. 〇: ammonium nitrogen treatment, ■: nitrate nitrogen treatment. * indicates a significant difference at the 5% level. Error bars represent standard errors

Figure 2 shows the temporal changes in total root length. Total root length increased exponentially with DAS under both NH₄-N and NO₃-N treatments. Up to 30 DAS, there were no significant differences between the N treatments (*p* > 0.05). However, from 40 DAS onward, total root length in the NH₄-N treatment became significantly greater than that in the NO₃-N treatment (*p* < 0.05).

**Fig. 3:**
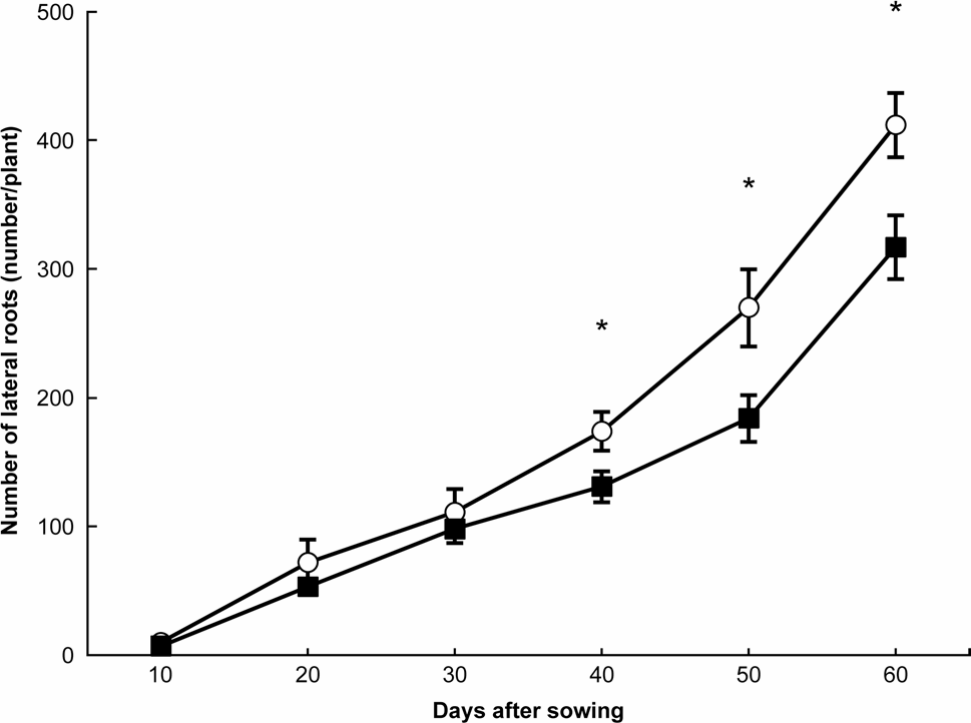
Changes in the number of lateral roots under each treatment. 〇: ammonium nitrogen treatment, ■: nitrate nitrogen treatment. * indicates a significant difference at the 5% level. Error bars represent standard errors

The number of lateral roots exhibited a trend similar to total root length under both N treatments (Fig. 3). From 40 DAS onward, the NH₄-N treatment resulted in a significantly greater number of lateral roots than the NO₃-N treatment (*p* < 0.05).

**Fig. 4:**
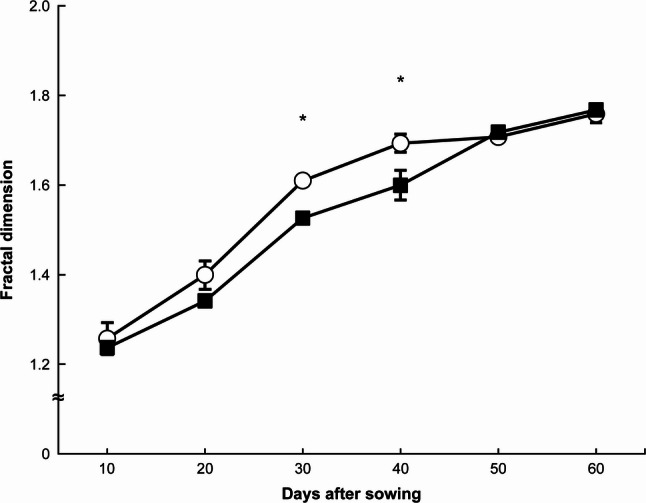
Changes in fractal dimension under each treatment. 〇: ammonium nitrogen treatment, ■: nitrate nitrogen treatment. * indicates a significant difference at the 5% level. Error bars represent standard errors

Figure 4 presents the temporal changes in D. Under both N treatments, D increased approximately linearly with DAS. During the mid-growth stages (30 and 40 DAS), D was significantly higher under the NH₄-N treatment than under the NO₃-N treatment. However, no significant differences were observed during the early (10 and 20 DAS) or late growth stages (50 and 60 DAS) (*p* > 0.05).

**Fig. 5:**
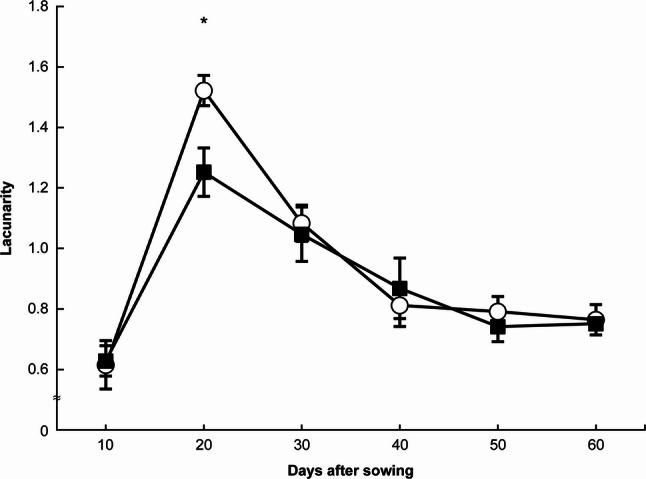
Changes in lacunarity under each treatment. 〇: ammonium nitrogen treatment, ■: nitrate nitrogen treatment. * indicates a significant difference at the 5% level. Error bars represent standard errors

Figure 5 shows the temporal changes in Lf. Lf increased rapidly from 10 to 20 DAS under both N treatments and then gradually decreased with further plant growth. At 20 DAS, the NH₄-N treatment showed a significantly higher Lf than the NO₃-N treatment (*p* < 0.05); however, no significant differences were observed at other time points (*p* > 0.05).

**Fig. 6:**
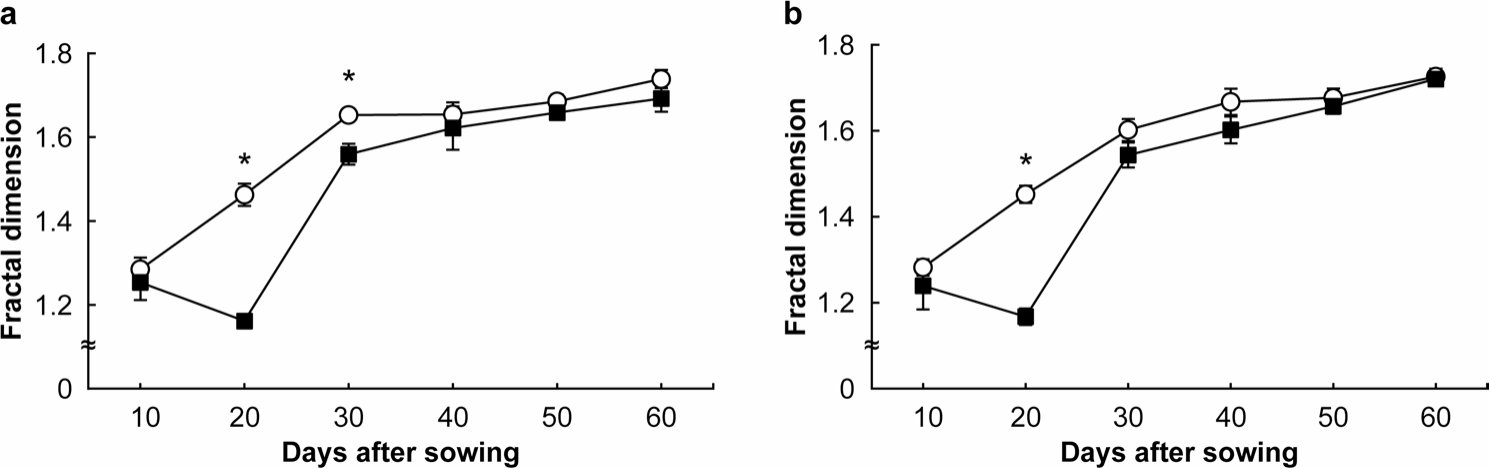
Changes in the fractal dimension of the upper and lower root systems under different treatments. **a**: ammonium nitrogen treatment, **b**: nitrate nitrogen treatment. 〇: upper root system, ■: lower root system. * significant difference at *p* < 0.05. Error bars represent standard error.

Figure 6 shows the temporal changes in D for the upper and lower regions of the images. D in the upper region exhibited an increasing trend with plant growth under both NH₄-N and NO₃-N treatments. In contrast, D in the lower region decreased from 10 to 20 DAS under both N treatments, then shifted to an increasing trend thereafter. Under the NH₄-N treatment, D in the upper region was significantly higher than in the lower region at 20 and 30 DAS, whereas under the NO₃-N treatment, a significant difference between the two regions was detected only at 20 DAS (*p* < 0.05). At other time points, no significant differences between the upper and lower regions were observed for either treatment (*p* > 0.05).

**Fig. 7:**
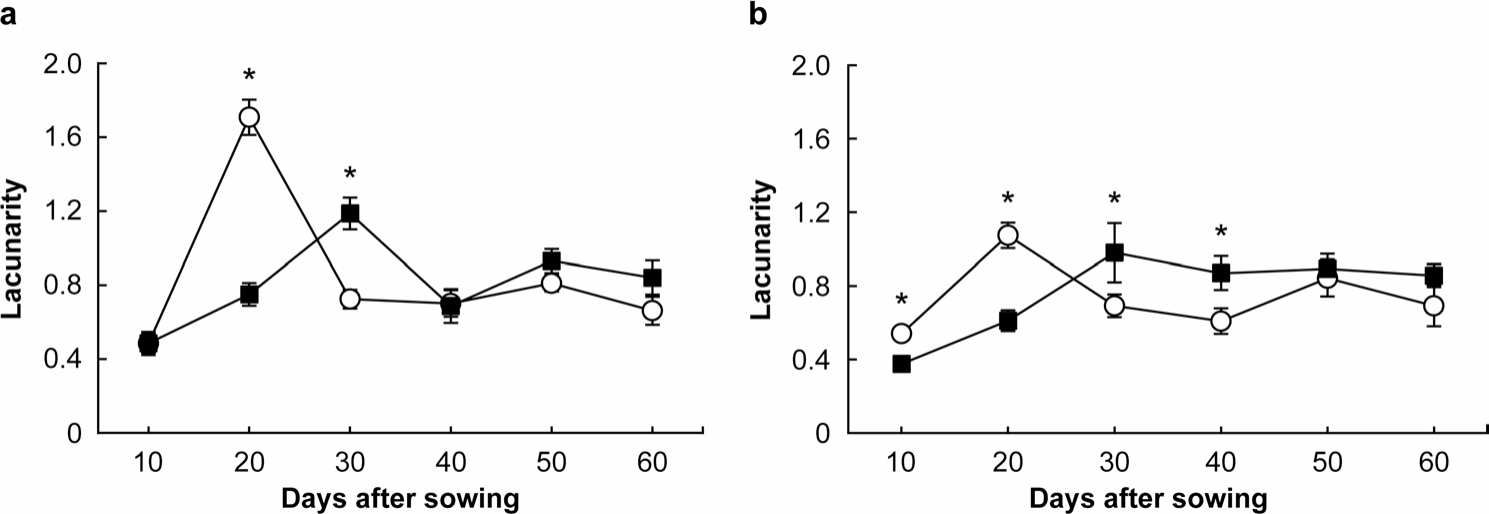
Changes in the lacunarity of the upper and lower root systems under different treatments. **a**: ammonium nitrogen treatment, **b**: nitrate nitrogen treatment. 〇: upper root system, ■: lower root system. * significant difference at p < 0.05. Error bars represent standard error

Figure 7 shows the temporal changes in Lf for the upper and lower regions of the images. Lf in the upper region increased until 20 DAS under both NH₄-N and NO₃-N treatments, then decreased from 20 to 30 DAS. After 30 DAS, values fluctuated widely under both N treatments with no consistent trend. In contrast, Lf in the lower region increased until 30 DAS under both N treatments. Under the NH₄-N treatment, Lf decreased sharply from 30 to 40 DAS and then showed large fluctuations. Under the NO₃-N treatment, Lf gradually decreased after 30 DAS. In the NH₄-N treatment, Lf in the upper region was significantly higher than in the lower region at 20 DAS (*p* < 0.05), whereas at 30 DAS, the lower region showed a significantly higher value (*p* < 0.05). No significant differences between regions were found at other time points (*p* > 0.05). In the NO₃-N treatment, Lf in the upper region was significantly higher than in the lower region at 10 and 20 DAS (*p* < 0.05), while at 30 and 40 DAS, the opposite pattern was observed (*p* < 0.05). No significant differences between the two regions were detected at 50 and 60 DAS (*p* > 0.05).

**Fig. 8:**
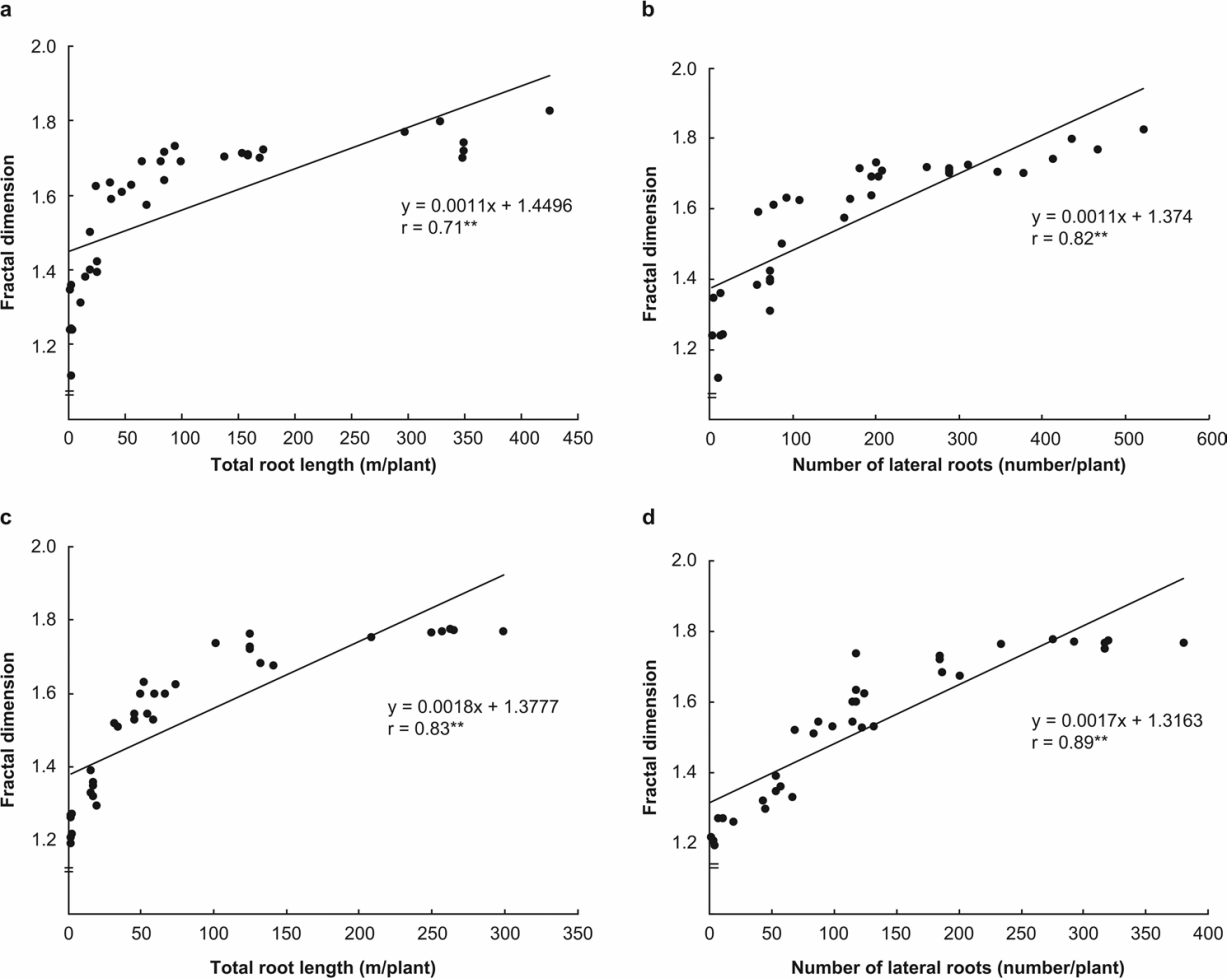
Relationship between fractal dimension and total root length and number of lateral roots. **a**: relationship between fractal dimension and total root length in ammonium nitrogen treatment, **b**: relationship between fractal dimension and number of lateral roots in ammonium nitrogen treatment, **c**: relationship between fractal dimension and total root length in nitrate nitrogen treatment, **d**: relationship between fractal dimension and number of lateral roots in nitrate nitrogen treatment. The solid line represents the regression line. y and r represent the regression equation and correlation coefficient, respectively. ** indicates a significant difference at the 1% level

Figure 8 shows the relationship between D and total root length as well as the number of lateral roots. Under both NH₄-N and NO₃-N treatments, D exhibited a strong positive correlation with both total root length and the number of lateral roots. Furthermore, these differences were found to be statistically significant (*p* < 0.05).

Table 2 presents the correlation coefficients between Lf and D, total root length, and the number of lateral roots for 10–60 DAS (the entire experimental period), 10–20 DAS (the phase during which Lf increased), and 20–60 DAS (the phase during which Lf decreased). During 10–60 DAS, correlation coefficients between Lf and D, total root length, and lateral root number were low under both NH₄-N and NO₃-N treatments, with no significant differences observed (*p* > 0.05). During 10–20 DAS, Lf exhibited positive correlations with D, total root length, and lateral root number, and these correlations were statistically significant (*p* < 0.05 or *p* < 0.01). During 20–60 DAS, in contrast to the earlier period, Lf showed negative correlations with D, total root length, and lateral root number, and these relationships were also statistically significant (*p* < 0.05 or *p* < 0.01).

**Table 2:** Correlation coefficients between lacunarity and fractal dimension, total root length, and number of lateral roots from 10–60 DAS, 10–20 DAS, and 20–60 DAS

Parameter	10–60 DAS		10–20 DAS		20–60 DAS	
	NH₄-N treatment	NO₃-N treatment	NH₄-N treatment	NO₃-N treatment	NH₄-N treatment	NO₃-N treatment
L × D	−0.219 (ns)	−0.208 (ns)	0.584 (*)	0.742 (**)	−0.789 (**)	−0.621 (**)
L × TL	−0.317 (ns)	−0.290 (ns)	0.875 (**)	0.945 (**)	−0.455 (*)	−0.444 (*)
L × NL	−0.301 (ns)	−0.224 (ns)	0.942 (**)	0.916 (**)	−0.581 (**)	−0.446 (*)

*Abbreviations*: *NH₄-N* ammonium nitrogen, *NO₃-N* nitrate nitrogen, *L* lacunarity, *D* fractal dimension, *TL* total root length, *NL* number of lateral roots, *DAS* days after sowing

Significance: ns = not significant; * *p* < 0.05; ** *p* < 0.01 (Tukey’s test)

## Discussion

The results of this study demonstrated that the use of D and Lf can detect subtle differences in root system development caused by the chemical forms of applied N at earlier growth stages than total root length or the number of lateral roots (Figs. 2, 3, 4 and 5). Furthermore, the findings suggest that Lf is a useful indicator for identifying differences in root system development during the early growth stage, D during the middle stage, and total root length and lateral root number during the later stage.

D reflects the degree of root system complexity, with higher values corresponding to more complex structures [18]. In contrast, Lf values decrease when the root system is more homogeneous and increase when it is more heterogeneous [18, 19]. Accordingly, the root system under NH₄-N treatment, compared with that under NO₃-N treatment, showed no difference in complexity or homogeneity immediately after germination. However, it became more heterogeneous during the early growth stage (20 DAS) and subsequently developed a more complex structure during the mid-growth stage (30 and 40 DAS).

Observation of root system images revealed that at 10 DAS and 20 DAS, the number of lateral roots on the upper part of the taproot was greater than that on the lower part under both NH₄-N and NO₃-N treatments (Fig. 1). Previous studies have reported that in alfalfa, the root system initially develops through elongation of the taproot, followed by the sequential emergence of first-order lateral roots from the upper to the lower parts of the taproot [20]. Similar patterns of emergence have also been reported for second- and third-order lateral roots [20]. Based on the analysis of D in the upper and lower regions of the root system, both NH₄-N and NO₃-N treatments showed increasing complexity in the upper region as growth proceeded, while the lower region decreased in complexity from 10 DAS to 20 DAS and then increased thereafter (Fig. 6). In addition, homogenization progressed earlier in the upper root region than in the lower region under both N treatments (Fig. 7). These results indicate that the root development pattern observed in this study is consistent with earlier findings [20]. The high root system heterogeneity observed at 20 DAS in both N treatments (Fig. 5) was likely due to uneven lateral root development in the upper part of the taproot. At 30 DAS, although heterogeneity in the lower root zone increased under both N treatments relative to 20 DAS (Fig. 7), overall homogenization of the root system progressed (Fig. 5), possibly due to stronger homogenization in the upper region compared with the increased heterogeneity in the lower region (Fig. 7).

At 20 DAS, the root tip length (the distance from the tip of the taproot to the first primary lateral root appearing toward the base) was approximately 10.3 mm for the NH₄-N treatment and approximately 11.3 mm for the NO₃-N treatment. However, no significant difference between the two N treatments was observed at the 5% level. In alfalfa, it has been reported that primary lateral roots emerging from the taproot can develop multiple roots from the same position [20]. However, at 20 DAS in this experiment, only one primary lateral root emerged from each position under both the NH₄-N and NO₃-N treatments. Therefore, the observed difference in root system heterogeneity between the two N treatments at 20 DAS is suggested to have arisen partly from variation in the emergence of higher-order lateral roots beyond the secondary lateral roots.

In green beans, a negative correlation between Lf and D has been reported [7]. In this study, a similar relationship was observed during the period of Lf decline (20–60 DAS). However, during the phase of Lf increase (10–20 DAS) and throughout the entire experimental period (10–60 DAS), the relationship differed from that reported previously [7] (Table 2). Moreover, the relationship between Lf and total root length or lateral root number also varied depending on whether Lf was increasing, decreasing, or evaluated across the entire growth period (Table 2). This variation is likely because D, total root length, and lateral root number describe the developmental pattern of the root system itself, whereas Lf reflects the degree of variation among lateral roots, i.e., the gaps surrounding them [18]. In other words, such fluctuations in correlation may arise because Lf increases when taproot growth is active and lateral root development becomes uneven, irrespective of overall root system growth. Conversely, Lf decreases when taproot growth slows and lateral root development becomes more uniform.

In these experiments, we detected a time lag between the growth stages at which Lf differed between NH₄-N and NO₃-N treatments (i.e., 20 DAS) and those at which D differed (i.e., 30 and 40 DAS) (Figs. 4 and 5). Because D merely represents the complexity of root morphology without providing information on morphological type [18], this characteristic likely accounts for the observed time lag.

Another time lag was found between the stage (30 DAS) when D differed between NH₄-N and NO₃-N treatments and the stage (40 DAS) when total root length and lateral root number showed differences (Figs. 2, 3 and 4). This finding indicates that D is not simply a measure of root length or number but rather an index encompassing root system architecture and branching characteristics that cannot be expressed by those parameters alone [7, 8].

As previously reported [4], the NH₄-N treatment showed higher nitrogen uptake per unit time than the NO₃-N treatment during the early growth stage (25 DAS). Furthermore, at this stage, enzyme activities associated with root N uptake were also higher under NH₄-N treatment than under NO₃-N treatment [21]. At 20 DAS, root distribution patterns differed between NH₄-N and NO₃-N treatments (Fig. 5), suggesting a close relationship between N absorption and root system development.

In this study, roots were preserved in FAA solution after collection, which may explain why the measured values differed slightly from those obtained immediately after collection. However, since all samples were preserved under the same conditions, this is unlikely to have affected the overall trends or relationships in the results.

In addition, as noted previously [2–4], although the accompanying ion concentrations differed between NH₄-N and NO₃-N treatments, no growth impairment due to these ions was observed in any plant. Therefore, it is unlikely that accompanying ions influenced the outcomes.

In summary, the use of D and Lf enabled detection of detailed differences in root system development caused by the chemical form of N fertilizer at earlier growth stages than could be observed through root length or number alone. However, differences in alfalfa root length and number in response to the compound form of applied N are known to vary substantially depending on N concentration and medium pH [22, 23]. In addition, root morphology in alfalfa differs markedly among varieties and lineages [24, 25]. Moreover, results under soil cultivation may differ from those observed here because of soil-related effects. Therefore, to further evaluate the usefulness of D and Lf, future studies should investigate the influence of cultivation conditions and inter-varietal variation in greater detail.

## Conclusions

Using D and Lf revealed differences in root system development attributable to variations in the chemical forms of applied N that could not be detected through root length or root number alone. Furthermore, the findings suggest that these N-form-dependent differences in root development first appear in root distribution patterns, followed by changes in root system complexity, and ultimately in total root length and lateral root number.

## Data Availability

All data generated or analyzed during this study are included in this published article.

## Abbreviations

NH_4_-N: Ammonium nitrogen
NO_3_-N: Nitrate nitrogen
N: Nitrogen
DAS: Days after sowing
D: Fractal dimension
Lf: Lacunarity
